# Secreted Protein Acidic and Rich in Cysteine (SPARC) to Manage Coronavirus Disease-2019 (COVID-19) Pandemic and the Post-COVID-19 Health Crisis

**DOI:** 10.3390/medicines10050032

**Published:** 2023-05-16

**Authors:** Abdelaziz Ghanemi, Mayumi Yoshioka, Jonny St-Amand

**Affiliations:** 1 Department of Molecular Medicine, Faculty of Medicine, Laval University, Québec, QC G1V 0A6, Canada; abdelaziz.ghanemi.1@ulaval.ca; 2 Functional Genomics Laboratory, Endocrinology and Nephrology Axis, CHU de Québec-Université Laval Research Center, Québec, QC G1V 4G2, Canada; mayumi.yoshioka@crchudequebec.ulaval.ca

**Keywords:** secreted protein acidic and rich in cysteine (SPARC), coronavirus disease-2019 (COVID-19), potential therapies

## Abstract

Coronavirus disease-2019 (COVID-19) has had and will have impacts on public health and health system expenses. Indeed, not only it has led to high numbers of confirmed COVID-19 cases and hospitalizations, but its consequences will remain even after the end of the COVID-19 crisis. Therefore, therapeutic options are required to both tackle the COVID-19 crisis and manage its consequences during the post COVID-19 era. Secreted protein acidic and rich in cysteine (SPARC) is a biomolecule that is associated with various properties and functions that situate it as a candidate which may be used to prevent, treat and manage COVID-19 as well as the post-COVID-19-era health problems. This paper highlights how SPARC could be of such therapeutic use.

Coronavirus disease-2019 (COVID-19) can been considered as the greatest health crisis which humanity has faced since the Spanish flu pandemic [1] in terms of the number of deaths. In addition, the resulting saturation of the health system and socio-economic consequences are also key impacts of the COVID-19 crisis [2,3] resulting from the applied measures aiming to limit COVID-19’s spread. Ironically, such measures (including confinement [4,5,6] and work-from-home policies [7,8,9,10])) have negative impacts on health and could even increase vulnerability to COVID-19 by promoting COVID-19 risk factors (obesity [11,12], physical inactivity [13,14], unhealthy diet, etc.) within the population. Indeed, one of the interesting characteristics of this context is the fact that not only do these risk factors increase COVID-19’s infectiousness and severity, but the measures applied by governments and health authorities to limit COVID-19’s spread also worsen these risk factors. For instance, individuals are confined at home and develop a new lifestyle [15,16], have reduced physical activity, tend towards sedentariness (beyond physical inactivity [17]), and work from home (cognitive work) [18], which can induce glycemic instability [19], cause individuals to consume more food (quantity), consume more junk food (poor quality), have reduced social interactions and less social activities and develop mental health (risk of psychiatric illness) problems [20,21,22,23,24]. All these elements can impact the metabolic profile, diet behavior and obesity development. They can also lead to smoking, alcohol consumption and drug use, which are additional risk factors for various health problems. Such mechanistic links extend in multiple directions. Whereas they can impact obesity development [25] and diet behavior (emotional eating) [26,27,28,29,30], diet [31] and obesity [32] can also affect mental health, including self-esteem [33]. Importantly, the different diseases that can develop with obesity (as their risk factor), such as coronary heart disease, type 2 diabetes, liver disease and cancers [34,35,36,37,38,39,40,41,42,43], will further place individuals at risk of developing severe forms of COVID-19 [44,45,46].

Moreover, physical inactivity is associated with impaired immunity [47] and increased systemic inflammation [48], which are more important to highlight during this ongoing COVID-19 crisis [47]. As illustrated by the examples given above, obesity increases susceptibility to COVID-19, which can be corrected through exercise and a controlled diet [49,50,51,52,53,54,55,56,57,58]. However, at the same time, the COVID-19-crisis-related measures taken by different governments are oriented towards inducing a novel socioeconomic environment. This environment will lead to obesity by increasing food intake, physical inactivity and even reduced food quality, since the economic situation renders healthy food hardly affordable. In conclusion, to address such factors that increase susceptibility to, and risk of, COVID-19, beyond targeting these factors, we must also think of innovative solutions to modify or change the measures taken to limit COVID-19’s impacts in a way that could reduce obesity-favoring COVID-19-crisis-related factors.

Among the most noticeable COVID-19-severity-determining factors is inflammation. Indeed, the severity of COVID-19 depends on the inflammatory reactions that develop as a response to the virus. Based on what is known about the inflammatory storm in severe COVID-19 cases [59,60,61,62,63], we can distinguish numerous molecular mediators and cellular factors, such as interleukins [64]. Other cytokines could be explored for therapeutic usage. For instance, interferon beta-1a has led to clinical improvement in hospitalized COVID-19 patients [65], which highlights interferon beta-1a as a potential therapy for COVID-19 [66,67]. Therefore, one of the approaches could be to modify the cytokines in order to reduce the inflammatory-induced damage, optimize antiviral immune functions and maintain the homeostasis (metabolic, regeneration, hormonal, etc.) of the vital tissues throughout COVID-19’s evolution. 

Diverse diseases, pathological conditions and health problems worsen COVID-19 prognosis. These include two of the major health problems of our modern societies: obesity and ageing. Indeed, obese and ageing subjects have a chronic inflammation status [68,69] that is considered as a “basal status” upon which COVID-19 builds further inflammatory phenomena, thus explaining why obese patients are more susceptible to COVID-19, especially given the impacts of physical inactivity and sedentary behavior in the case of autoimmune diseases [70]. All these elements indicate an over-“stimulation” of the immune system even before COVID-19 is established. These elements are the key characteristics of COVID-19-crisis-related health consequences. 

On the other hand, the post-COVID-19 era represents an additional health challenge for the general population and not only for those infected with severe acute respiratory syndrome coronavirus-2 (SARS-CoV-2). The post-COVID-19 era will bring challenges that will add to the pre-existing healthcare system problems, including post-intensive care syndrome, reduced healthcare for non-COVID-19 patients and other diseases that may worsen during COVID-19.

Those infected with SARS-CoV-2 may be affected by COVID-19 consequences on their health, such as fatigue and shortness of breath [71], which will limit their ability to perform the required level of physical activity. Therefore, these patients need, for instance, an “exercise substitute”. In addition, the general population will still be affected by the consequences of confinement and the economic crisis in terms of their health, such as those resulting from a sedentary lifestyle and confinement, including obesity [72], cardiovascular diseases [73], diabetes [74] and possible immunity decline [47]. 

Therefore, there is a need for effective approaches to tackle this multi-level crisis, especially with the limited number of approved therapeutic options and the possibly limited impacts of the developed vaccines. It remains crucial to find new therapeutic tools and possible interventions. In light of this, the exercise-induced protein known as secreted protein acidic and rich in cysteine (SPARC) emerges as a potential therapeutic option. *SPARC*/*Sparc* was initially identified as an exercise-induced gene, both in vivo and in vitro, following functional genomics explorations [75,76]. Studies have suggested that SPARC is responsible for exercise-induced muscle phenotype changes [77] and that it might mediate exercise-induced effects [78,79]. Thus, SPARC could be an “exercise substitute” [79]. 

Importantly, the properties mediated and the effects induced by SPARC suggest that the induction of SPARC administration/expression could counteract risk factors for severe forms of COVID-19, limit the impacts of the pandemic measures during the post-COVID-19 era and even form part of the therapies used for hospitalized COVID-19 patients. Among SPARC’s properties that could lead to possible therapeutic applications, we have anti-inflammatory [80], anti-ageing [78,79], anti-sarcopenia/muscle atrophy [81] and anti-obesity properties [82], as well as SPARC’s importance for immunity [83]. The potential anticancer property of SPARC is also worth mentioning in this context [82,84], since cancer might increase the risk of COVID-19 adverse outcomes [46,85,86,87]. Thus, SPARC reverses numerous negative impacts of obesity, in addition to other factors, such ageing and diverse obesity-related and age-related health conditions, that render individuals more susceptible to COVID-19. 

SPARC, due to its various properties, might constitute an efficient therapeutic tool, since it acts on various levels (Figure 1) that would allow it to tackle health problems related to COVID-19 severe-form risk factors, as well as public health problems during the post-COVID-19 era, and to help COVID-19 patients both during hospitalization and in the management of long COVID-19 (complications following acute illness).

Exercise remains a strong option in the context of the COVID-19 crisis as well as the post-COVID-19 era. However, with many individuals being unable (disability, hospitalization, etc.) or unwilling (mental health) to perform the required physical activity, SPARC could emerge as an alternative [79] or as an additional therapeutic tool worth exploring to tackle COVID-19 and its consequences on various levels. 

According to the different pathways at the molecular and cellular levels linking SPARC to exercise, the resulting phenotypic changes will involve increasing myogenesis, the extracellular matrix and muscle glucose transporter expression, as well as decreasing adipogenesis and mitochondrial dysfunction [88]. The exploration of such pathways represents a starting point towards the development of novel therapeutic options for COVID-19 and its complications, which are urgently required, especially with the risk of the emergence of COVID-19 variants [89,90,91,92].

## Figures and Tables

**Figure 1:**
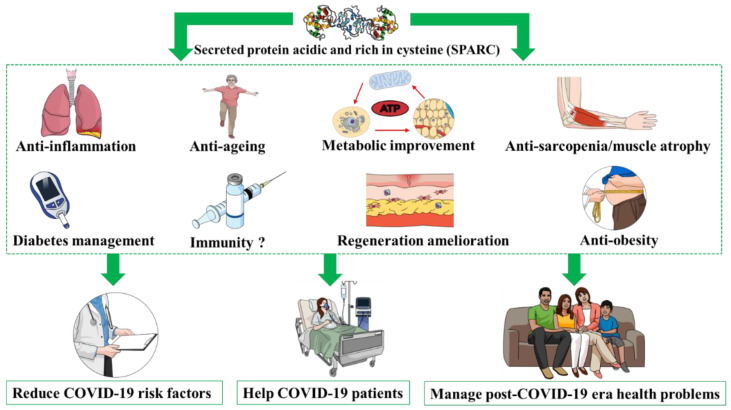
Properties of secreted protein acidic and rich in cysteine (SPARC) as a potential therapeutic option to manage coronavirus-disease-2019 (COVID-19)-related health consequences on various levels.
